# Feasibility of artificial intelligence-assisted fast magnetic resonance imaging technology in the ankle joint injury: a comparison of the proton density-weighted image

**DOI:** 10.3389/fradi.2025.1673619

**Published:** 2025-10-24

**Authors:** Sihan Xu, Wenjuan Cao, Luyi Wang, Pangxing Guo, Yuhai Cao, Honghai Chen

**Affiliations:** Radiology Department, The Second Hospital of Dalian Medical University, Dalian, China

**Keywords:** ankle joint, magnetic resonance imaging, artificial intelligent, image quality, diagnostic efficacy

## Abstract

**Objective:**

To evaluate the image quality and diagnostic efficacy of proton density-weighted MRI with intelligent quick magnetic resonance (iQMR) technology in the ankle joint injury.

**Materials and methods:**

Forty-six patients with ankle injuries were prospectively enrolled, and proton density-weighted fat suppression imaging was performed on a 3.0T MRI scanner using both an iQMR protocol (48.28 s) and a Conventional protocol (113.00 s), respectively. The original image was processed using iQMR to improve spatial resolution and reduce noise interference. Thus, four sets of images (iQMR raw, iQMR-processed, Conventional raw, and Conventional-processed) were generated. Image quality and diagnostic efficacy were assessed by objective metrics (signal-to-noise ratio, SNR and contrast-to-noise ratio, CNR), subjective scores (tissue edge clarity/sharpness, signal uniformity, fat suppression uniformity, vascular pulsation artifacts, and overall image quality), and ligaments/tendons injury grade.

**Results:**

The SNRs (tibia, talus, etc.) and CNRs (talus-flexor hallucis longus, etc.) of iQMR-processed images were significantly higher than those of Conventional raw images (*P* < 0.05), except for the SNR of Achilles tendon (*P* > 0.05). And the iQMR-processed images were superior to the Conventional raw images in the scores of edge clarity/sharpness, signal uniformity and overall image quality (*P* < 0.05), with no significant differences in fat suppression uniformity and vascular pulsation artifacts (*P* > 0.05). There was no significant difference among the four groups of images in ligaments/tendons injury grading (*P* > 0.05), but the iQMR-processed images improved diagnostic confidence [*κ* (kappa) = 0.919].

**Conclusion:**

The iQMR technology can effectively shorten the scan time, improve the image quality without affecting the diagnostic accuracy, which is especially suitable for the motion artifacts-sensitive patients and optimizes clinical workflow.

## 1 Introduction

The ankle joint, a composite structure formed by the articular surfaces of the distal tibia, distal fibula, and the talar trochlea, serves as the primary weight-bearing joint critical for maintaining upright posture and facilitating gait. Injuries to this joint are clinically prevalent and frequently lead to chronic pain, impaired mobility and diminished quality of life (1–3). Current diagnostic imaging for ankle pathologies predominantly relies on radiography and computed tomography (CT). However, these modalities exhibit limited soft tissue resolution, particularly for cartilage and ligaments, which may compromise diagnostic accuracy in cases of occult injuries (4, 5). Magnetic resonance imaging (MRI) has become the gold standard for evaluating osseous and soft tissue injuries owing to its exceptional soft tissue contrast and absence of ionizing radiation (6, 7). Nevertheless, traditional MRI sequences require prolonged acquisition times, which presents significant challenges for patients experiencing acute pain or swelling. Inability to remain motionless during scanning often introduces motion artifacts, degrading image quality and compromising diagnostic accuracy (8, 9). Therefore, reducing MRI acquisition time has emerged as a critical research priority in medical imaging, aiming to enhance patient compliance, minimize motion-induced artifacts and improve clinical diagnostic efficacy (10–12).

In clinical MRI, Parallel Imaging (PI) and Compressed Sensing (CS) have been widely used to accelerate scanning (13). However, both methods face inherent limitations: PI's acceleration capability is fundamentally constrained by coil geometry, invariably leading to signal-to-noise ratio (SNR) degradation, whereas CS relies on sparsity assumptions that are prone to generate nonlinear reconstruction artifacts, potentially compromising diagnostic reliability (14, 15).

**Figure 1:**
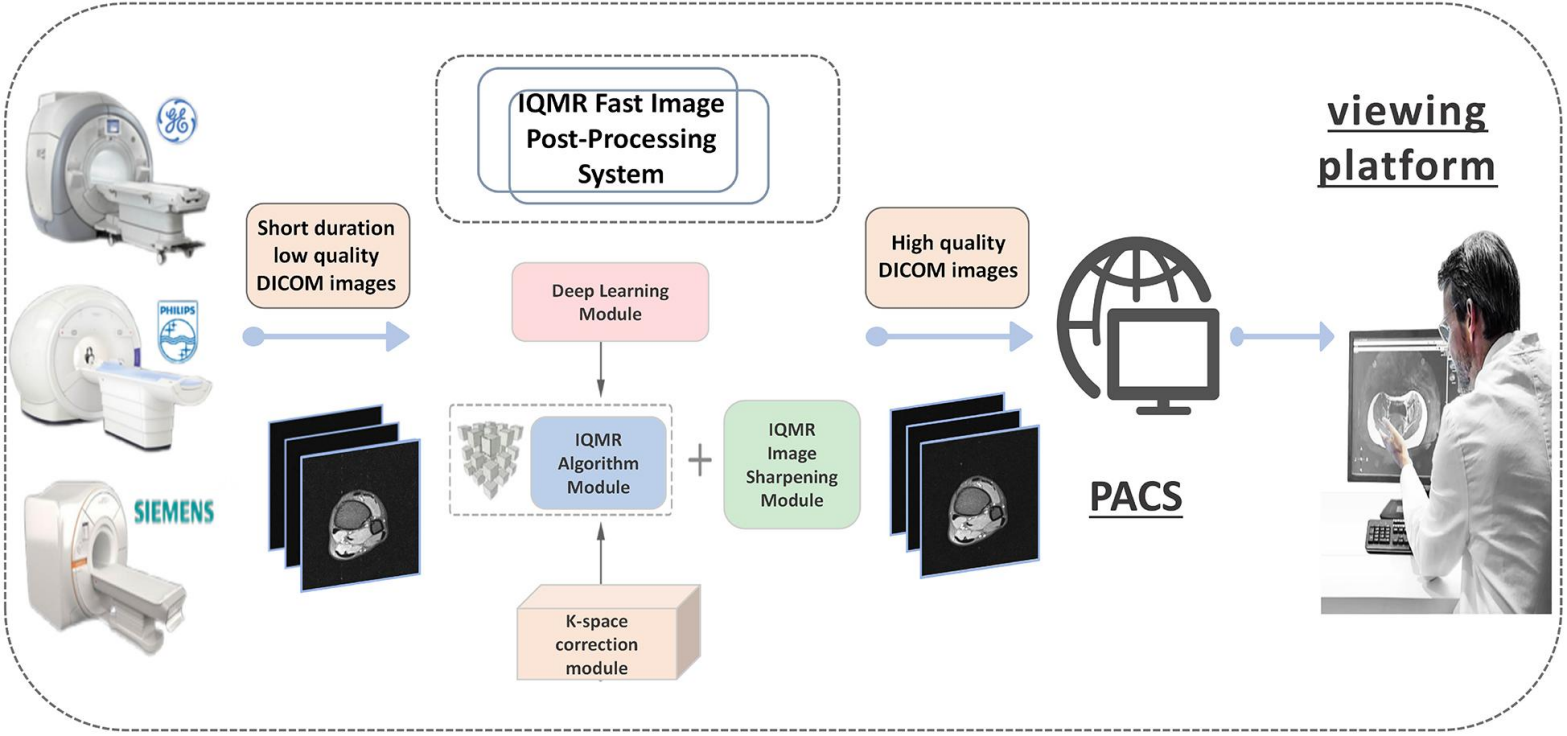
Research flowchart. iQMR, intelligent quick magnetic resonance.

Recent advances in artificial intelligence (AI) have catalyzed the development of intelligent quick magnetic resonance (iQMR), an end-to-end deep learning-based reconstruction system designed to overcome these challenges (16, 17). The iQMR platform integrates three dedicated modules: a deep learning reconstruction algorithm; an iterative reconstruction processor; and a k-space correction unit, collectively optimizing the image reconstruction workflow (15, 18–20) (Figure 1). Compatible with major MRI vendors (Siemens, GE and Philips), the iQMR system utilizes hospital-grade servers and seamlessly integrates into the DICOM workflow between MRI scanners and Picture Archiving and Communication Systems (PACS). The system automates the complete processing pipeline, from raw data acquisition to high-fidelity image reconstruction and distribution, while preserving existing clinical workflows and delivering diagnostically superior image quality (21). However, research on the clinical feasibility of fast ankle MRI strategies using iQMR is still rare to our knowledge (22, 23).

The iQMR technology integrates multiple algorithmic modules, with the Iterative Image Reconstruction (IIR) module and the Image Sharpening module serving as the core components that collectively perform the primary enhancement tasks. The IIR module is a post-processing algorithm based on volumetric data, designed primarily for the retrospective reconstruction of high-noise MRI images. This algorithm significantly reduces image noise, recovers anatomical details obscured by noise, and improves image quality parameters such as edge enhancement. The processing pipeline begins by decomposing the input MRI dataset into multiple three-dimensional blocks. Multidimensional features are computed for each block, which are then mapped into a feature space and grouped based on specific similarity metrics. Leveraging the similarity relationships between blocks and noise statistical priors, the algorithm performs joint prediction and separation of the signal and noise. This procedure iterates until predefined convergence criteria are met. Subsequently, specific filters are applied to enhance image features (e.g., edge structures) and tailor the reconstructed image to better align with radiologists' visual preferences. Finally, the dataset can be reconstructed into images along any orientation (axial, sagittal, or coronal) and with a specified slice thickness as required clinically. The core of the algorithm is tunable via multiple parameters, allowing control over output characteristics such as overall smoothness, sharpness level, and edge enhancement intensity. Furthermore, an integrated machine learning module can automatically identify the optimal parameter combination for the input image and feed it into the iterative reconstruction pipeline to achieve the highest quality output.

Building upon the foundation laid by the IIR module, the iQMR Image Sharpening module further augments the image enhancement capabilities by specifically increasing sharpness and clarity. This module employs a fixed-parameter Convolutional Neural Network (CNN) that performs a deterministic nonlinear filtering operation on the input image. The image data is sequentially processed through a bank of filters composed of thresholding and scaled transformation operations. This process enhances the image's sharpness and clarity while significantly improving the visibility of fine details. The parameters for the iQMR sharpening filter were obtained through an image-guided optimization process. This process utilized paired high-resolution and low-resolution images to optimize the filter weights. The CNN architecture is based on a modified Super-Resolution Generative Adversarial Network (SRGAN), incorporating an adapted filter block structure and loss function. After training, this sharpening model can restore a low-resolution input image to a high-resolution, sharper output. The training leveraged a large-scale, multi-center MRI dataset comprising over 500,000 images from various mainstream scanner models and multiple hospitals. The dataset encompasses a wide range of clinical indications, magnetic field strengths, image qualities, tissue contrasts, acquisition parameters, and patient anatomies to ensure the model's broad applicability.

Upon completion of training, the model's weights, architecture, and all parameters are fixed, enabling the algorithm to operate as a stable nonlinear filtering system. This strategy ensures consistent and reliable performance across different datasets. Following the initial training phase, the model can be deployed without requiring further training or fine-tuning.

This study was designed to systematically evaluate the clinical utility of iQMR in ankle MRI by comparing its performance with conventional scanning protocol across three critical parameters: scanning efficiency, image quality, and diagnostic accuracy.

## 2 Materials and methods

### 2.1 Participants

**Figure 2:**
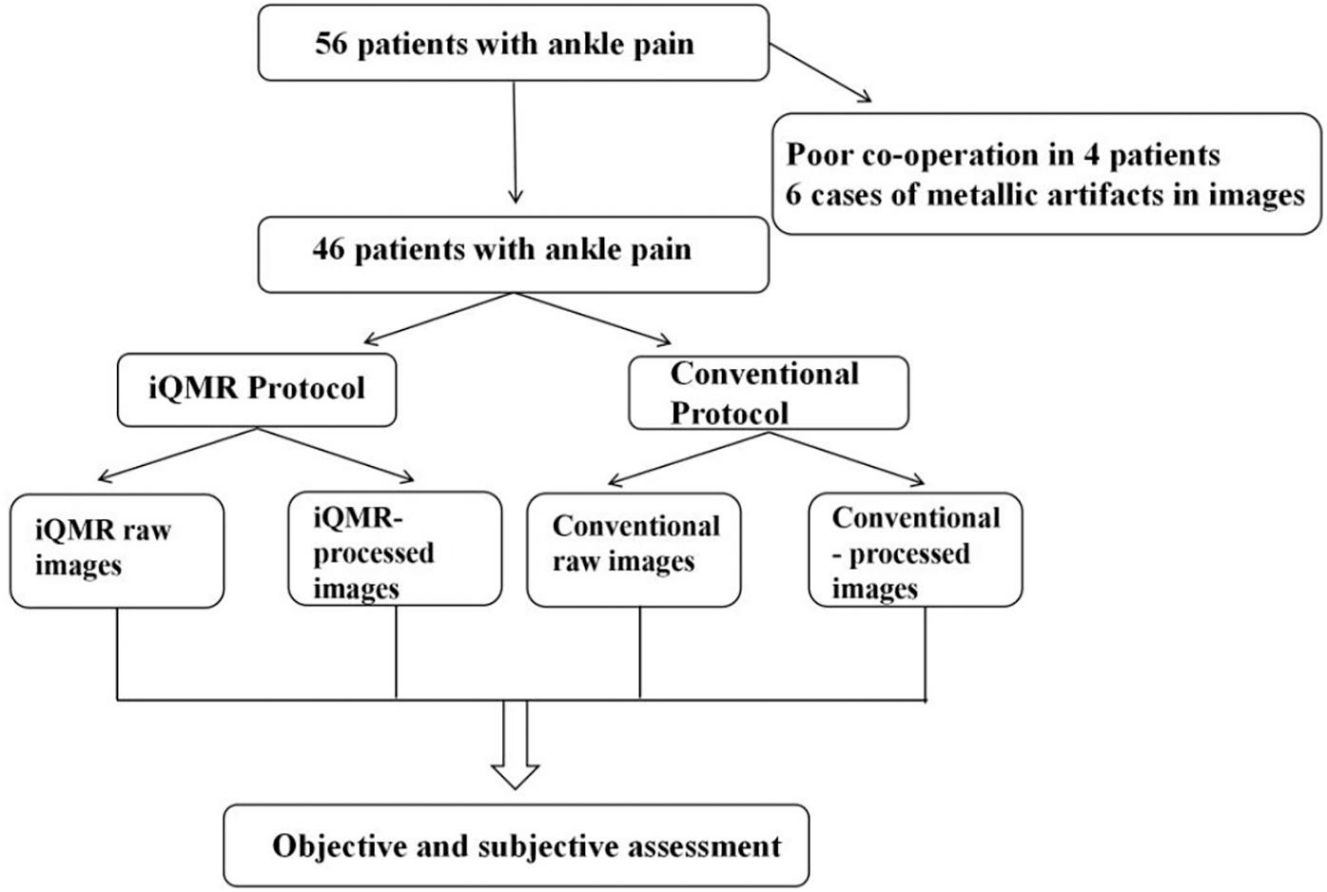
Schematic diagram illustrating the iQMR post-processing workflow. iQMR, intelligent quick magnetic resonance.

The prospective study was reviewed and approved by the Ethics Committee of the Second Affiliated Hospital of Dalian Medical University, and adhered to the principles of the Declaration of Helsinki. Written informed consent was obtained from all participants prior to enrollment. Fifty-six patients who underwent MRI examinations for clinically suspected ankle injuries from October 2024 to February 2025 at the Second Hospital of Dalian Medical University were enrolled. Inclusion criteria: (1) ankle injury; (2) no standard MRI contraindications; (3) age >= 18 years. Exclusion criteria: (1) history of ankle joint surgery within the preceding six months; (2) ferromagnetic implants in the ankle region; (3) incomplete MRI scans due to patient intolerance; (4) significant image artifacts affecting diagnostic assessment. Finally, 46 patients (17 males and 29 females; age range, 18–68 years) were included in our study (Figure 2). For each participant, we collected a range of data including demographic and clinical information, medical history, as well as MRI data.

### 2.2 Scan protocols

**Figure 3:**
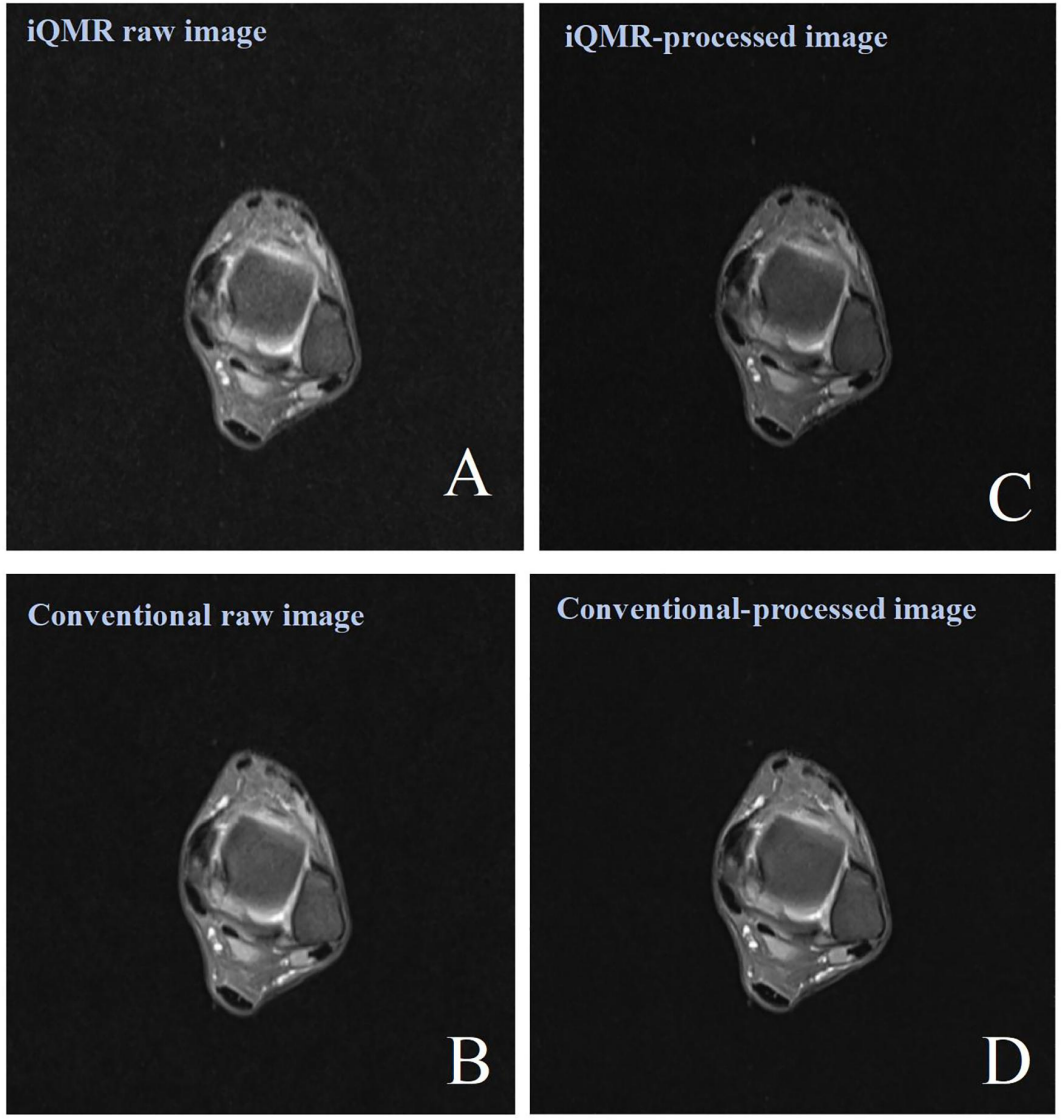
Female, 38 years old, patient with ankle pain. **(A)** iQMR raw PDWI-FS image; **(B)** Conventional raw PDWI-FS image; **(C)** iQMR-processed PDWI-FS image; **(D)** Conventional-processed PDWI-FS image. iQMR, intelligent quick magnetic resonance.

MRI examinations were performed using a 3.0T MRI scanner (Verio, Siemens Healthineers, Erlangen, Germany) equipped with a cranial phased array coil. To minimize motion artifacts, participants assumed the standard supine position with the affected ankle stabilized using foam padding and sandbags to restrict involuntary movement. The scanning plane was oriented along the anatomical axial plane, centered at the midpoint between the medial malleolus and lateral malleolus. All participants underwent a transverse-axis proton density-weighted fat saturation imaging (PDWI-FS) scan using an iQMR (acquisition time of 48.28 s) and a Conventional (acquisition time of 113 s) protocols. The detailed MRI protocols are presented in Table 1. The raw images were automatically transferred to the iQMR post-processing system, which generated both iQMR-processed images and Conventional-processed images (Figures 3A–D).

**Table 1:** Acquisition parameters and scan times for iQMR and conventional protocols.

Protocol	TR	TE	Slice thickness	Dist. factor	FOV read	FOV phase	Base resolution	Phase resolution	Phase encoding direction	ETL	Averages	TA
iQMR	2,400 ms	28 ms	3.5 mm	0.7 mm	180 mm	100%	320 mm	208 mm	A-P	10	1	48.28 s
Conventional	2,400 ms	28 ms	3.5 mm	0.7 mm	180 mm	100%	320 mm	240 mm	A-P	10	2	1 min 53 s

iQMR, intelligent quick magnetic resonance; TR/TE, repetition time/echo time; FOV, field of view; ETL, echo train length; TA, time of acquisition.

### 2.3 Quantitative assessment

**Figure 4:**
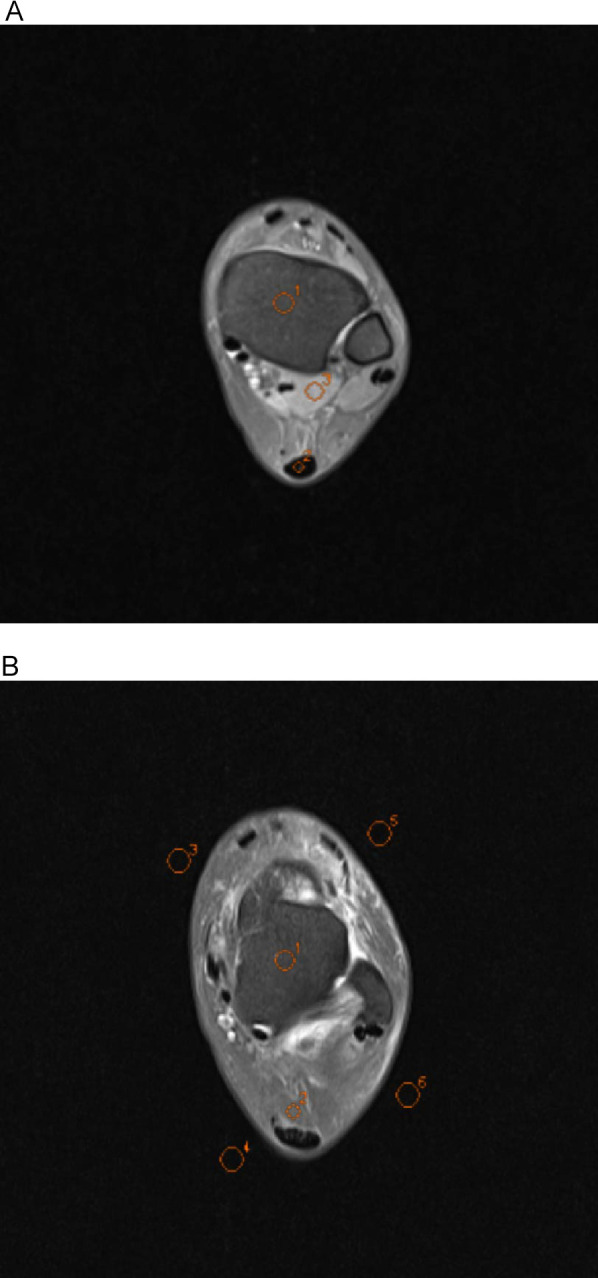
Axial ankle MR conventional raw images showing regions of interest (ROIs) used for quantitative analysis. All ROIs were manually delineated by a senior radiologic technologist (15 years of experience) under blinded conditions using the Siemens post-processing workstation. In image A, O1 indicates the tibia, O2 the Achilles tendon, and O3 the flexor hallucis longus; in image B, O1 represents the talus, O2 the Kager's fat pad, and O3–O6 the background regions for noise quantification.

A senior radiographer (15 years of experience) performed quantitative measurements on a Siemens post-processing workstation under blinded conditions (no access to subject data or sequence parameters). Regions of interest (ROIs) were manually placed in the following anatomical structures on three consecutive slices at identical levels, window widths, and window positions: tibia (20–30 mm^2^), talus (20–30 mm^2^), Achilles tendon (5–10 mm^2^), Kager's fat pad (10–20 mm^2^) and flexor hallucis longus (20–30 mm^2^). The mean signal intensity (SI) of each ROI was calculated based on three consecutive measurements. Image background noise was quantified by placing four ROIs (40–50 mm^2^) in the artifact-free corners of the image, with the final noise level defined as the mean standard deviation (SD) of these regions (Figures 4A,B). The SNR and contrast-to-noise ratio (CNR) were calculated using the following formulas:

$$\mathrm{SNR}=\frac{SI_{\mathrm{tissue}}}{SD_{\mathrm{contexts}}}$$

$$\mathrm{CNR}=\frac{\left|SI_{\mathrm{tissue}1}-SI_{\mathrm{tissue}2}\right|}{SD_{\mathrm{contexts}}}$$

### 2.4 Qualitative assessment

Two independent musculoskeletal radiologists (10 and 12 years of experience) conducted a double-blind assessment of four image sets. The evaluation dimensions included tissue edge clarity/sharpness, signal uniformity, fat suppression uniformity, vascular pulsation artifacts, and overall image quality. And the detailed scoring criteria using a 5-point Likert scale (range: 1 = “worst” to 5 = “best”) was as follows:

Score 1: Significant blurring of image edges, obvious signal non-uniformity, fat suppression failure, severe artifacts, no diagnostic value;
Score 2: Blurring of image edges visible, uneven signal, poor fat suppression, obvious artifacts, limited diagnosis value;
Score 3: Image edges with fair clarity and sharpness, more uniform signals, fair fat suppression, moderate artifacts, basic diagnosis can be satisfied;
Score 4: Good image edge clarity and sharpness, more uniform signal, better fat suppression, mild artifacts, good diagnostic value;
Score 5: Good image edge clarity and sharpness, uniform signal, ideal fat suppression, no artifacts, best diagnostic value.

### 2.5 Diagnostic assessment

The structures of ligaments and tendons were independently evaluated by the two musculoskeletal radiologists according to the Schweitzer classification system (24). The grading criteria was as follows: Grade 0: structurally intact with normal morphology and signal; Grade 1: post-traumatic fibrous changes (e.g., thickening or degenerative changes); and Grade 2: partial or complete tear. To further evaluate the practical value of iQMR technology in clinical diagnosis, the two aforementioned musculoskeletal radiologists independently performed diagnostic confidence ratings for key ligament and tendon structures under double-blind conditions. The assessments were conducted on four sets of ankle joint images: iQMR raw images, iQMR-processed images, conventional raw images, and conventional-processed images. The evaluated structures included the anterior talofibular ligament, posterior talofibular ligament, calcaneofibular ligament, Achilles tendon, posterior tibial tendon, flexor digitorum longus tendon, flexor hallucis longus tendon, peroneus brevis tendon, and peroneus longus tendon. A 5-point Likert scale was used for scoring, where 1 indicated very low confidence, 2 indicated low confidence, 3 indicated moderate confidence, 4 indicated high confidence, and 5 indicated very high confidence.

### 2.6 Statistical analysis

Normally distributed continuous variables are reported as Mean ± SD. Continuous variables were compared using one-way ANOVA under the assumption of homogeneity of variances (verified via Levene's test). For datasets violating this assumption, Welch's ANOVA was employed. *Post-hoc* pairwise comparisons utilized Tukey's test (equal variances) or Games-Howell test (unequal variances). To evaluate the agreement and potential bias in quantitative image quality parameters (SNR and CNR) between iQMR-processed images and conventional raw images, Bland-Altman analysis was performed on measurements from various anatomical structures (e.g., tibia, talus, Achilles tendon, Kager's fat pad, flexor hallucis longus muscle). The limits of agreement (LoA) and mean bias were calculated, and corresponding Bland-Altman plots were generated. Non-normally distributed variables are expressed as median with inter-quartile range [*M* (*Q*1, *Q*3)]. Between-group differences were assessed using the Friedman rank-sum test for repeated measures, followed by Bonferroni-corrected pairwise comparisons. Inter-rater reliability for qualitative scores was evaluated using weighted Cohen's kappa (*κ*), with *κ* values interpreted as follows: 0.81–1.00, excellent agreement; 0.61–0.80, substantial agreement; 0.41–0.60, moderate agreement. Subjective image quality scores and diagnostic confidence score (ordinal data) were compared across groups using the Friedman test, with Bonferroni-correction for *post-hoc* pairwise analysis. Ligaments/tendons injury grading (non-parametric categorical data) was analyzed via the Kruskal–Wallis test. Statistical significance was set at *P* < 0.05 for all tests. Data analysis was performed using IBM SPSS Statistics 27.0.

## 3 Results

### 3.1 Participant characteristics

A total of 46 patients (17 males and 29 females; age, 35.5 ± 14.5 years; age range, 18–68 years) with ankle injuries were included in this study.

### 3.2 Results of quantitative assessment

**Figure 5:**
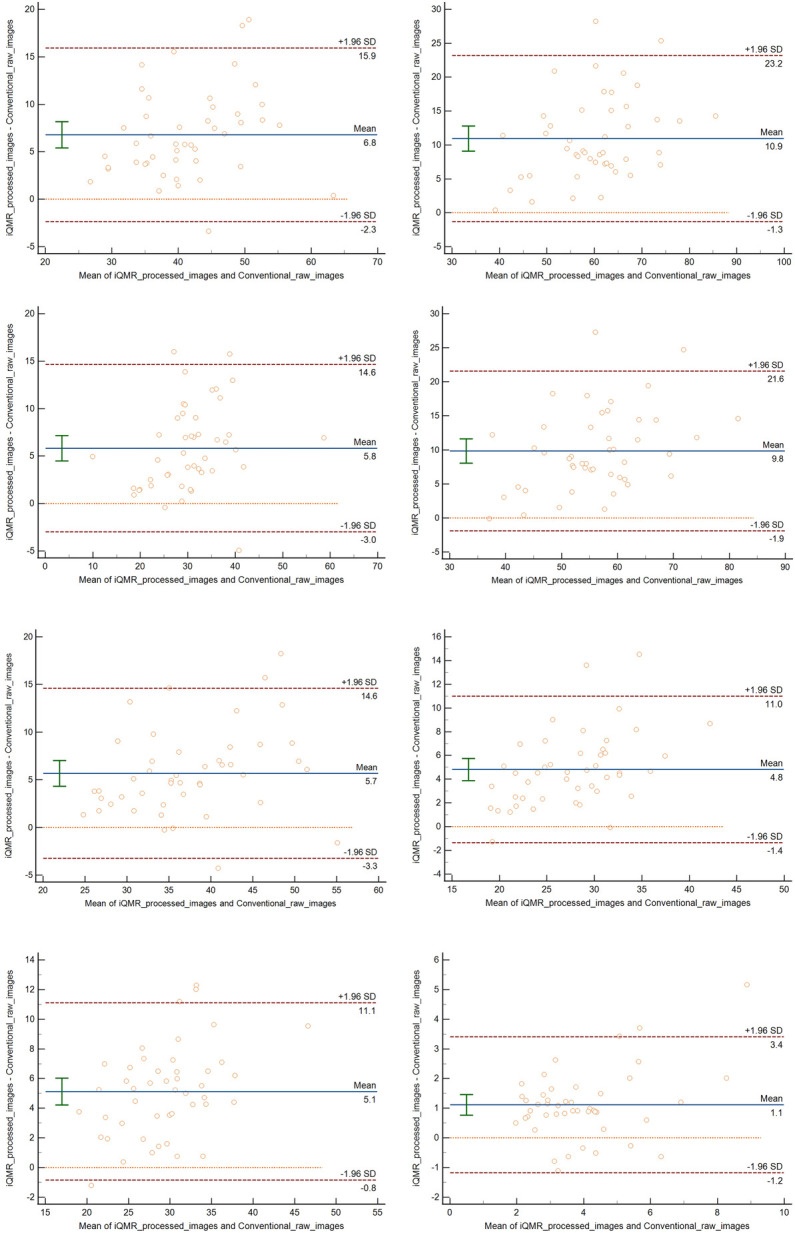
Bland–altman analysis revealed that the differences in SNR and CNR across all anatomical structures were predominantly concentrated within the agreement limits, with the mean bias approximating zero, indicating no substantial systematic bias or evident dispersion trend.

The ANOVA showed significant differences in all the SNRs and CNRs across the four groups of images (*P* < 0.001). *Post-hoc* pairwise comparisons showed that tibia SNR, talus SNR, Kager's fat pad SNR and flexor hallucis longus SNR, talus-flexor hallucis longus CNR, Achilles tendon-flexor hallucis longus CNR, Kager's fat pad-Achilles tendon CNR of the PDWI-FS sequences were statistically significant for each sequence (*P* < 0.001). The differences in Achilles tendon SNR between iQMR raw and iQMR-processed sequences, iQMR raw and Conventional-processed sequences, iQMR-processed and Conventional raw sequences, and Conventional raw and Conventional-processed sequences were all statistically significant (*P* < 0.05). And the differences in Achilles tendon SNR between iQMR raw and Conventional raw sequences, and iQMR-processed and Conventional-processed sequences were not statistically significant (*P* > 0.05). Detailed results are summarized in Table 2. Bland-Altman analysis revealed that the differences in SNR and CNR across all anatomical structures were predominantly concentrated within the agreement limits, with the mean bias approximating zero, indicating no substantial systematic bias or evident dispersion trend (Figure 5).

**Table 2:** Quantitative assessment results.

Items	iQMR raw sequences	iQMRp sequences	Conventional raw sequences	Conventionalp sequences	F-value	*P*-value
Tibia SNR	18.89 ± 3.84	30.07 ± 6.28	25.25 ± 4.85	38.92 ± 7.74	96.791	<0.001①②③④⑤⑥
Talus SNR	19.92 ± 3.77	31.93 ± 6.16	26.81 ± 4.93	41.56 ± 7.89	112.238	<0.001①②③④⑤⑥
Achilles tendon SNR	2.91 ± 1.21	4.50 ± 1.85	3.38 ± 1.45	5.42 ± 2.16	19.360	<0.001①③④⑥
Kager's fat pad SNR	28.19 ± 5.05	44.76 ± 8.68	37.98 ± 7.55	58.15 ± 11.51	108.387	<0.001①②③④⑤⑥
Flexor hallucis longus SNR	40.99 ± 6.74	65.41 ± 11.32	54.46 ± 9.23	82.96 ± 14.65	129.674	<0.001①②③④⑤⑥
Talus-Flexor hallucis longus CNR	21.06 ± 5.52	33.47 ± 8.79	27.65 ± 7.63	41.39 ± 11.75	48.428	<0.001①②③④⑤⑥
Achilles tendon-Flexor hallucis longus CNR	38.07 ± 6.36	60.91 ± 10.98	51.07 ± 8.78	77.54 ± 14.24	123.906	<0.001①②③④⑤⑥
Kager's fat pad-Achilles tendon CNR	25.28 ± 4.84	40.26 ± 8.54	34.59 ± 7.29	52.72 ± 11.21	96.599	<0.001①②③④⑤⑥

iQMR, intelligent quick magnetic resonance; iQMR_p_, iQMR-processed images; Conventional_p_, Conventional-processed images.

①Post hoc two-by-two comparison of iQMR raw with iQMR-processed PDWI-FS sequences; ②Post hoc two-by-two comparison of iQMR raw with Conventional raw PDWI-FS sequences; ③Post hoc two-by-two comparison of iQMR raw with Conventional-processed PDWI-FS sequences; ④Post hoc two-by-two comparison of iQMR-processed with Conventional raw PDWI-FS sequences; ⑤Post hoc two-by-two comparison of iQMR-processed with Conventional-processed PDWI-FS sequences; ⑥Post hoc two-by-two comparison of Conventional raw with Conventional-processed PDWI-FS sequences.

### 3.3 Results of qualitative assessment

The two musculoskeletal radiologists demonstrated excellent inter-rater agreement (weighted *κ* statistic = 0.871, CI: 0.896–0.846), with all subjective image quality scores ≥3, confirming diagnostic acceptability across sequences. Tissue edge clarity/sharpness: *κ* = 0.871 (95% CI: 0.924–0.818); Signal uniformity: *κ* = 0.871 (95% CI: 0.925–0.817); Fat suppression uniformity: *κ* = 0.854 (95% CI: 0.922–0.787); Vascular pulsation artifacts: *κ* = 0.821 (95% CI: 0.898–0.744); Overall image quality: *κ* = 0.867 (95% CI: 0.922–0.812). Friedman's test analysis revealed statistically significant differences among the four PDWI-FS sequences groups in tissue edge clarity/sharpness, signal uniformity, fat suppression uniformity, vascular pulsation artifacts, and overall image quality (*P* < 0.001). Tissue edge clarity/sharpness: Kendall's *W* = 0.915; Signal uniformity: Kendall's *W* = 0.888; Fat suppression uniformity: Kendall's *W* = 0.375; Vascular pulsation artifacts: Kendall's *W* = 0.457; Overall image quality: Kendall's *W* = 0.815. *Post-hoc* pairwise comparisons after Bonferroni correction identified specific inter-group variations, with detailed results summarized in Tables 3, 4.

**Table 3:** Qualitative scoring results.

Evaluation indicators	iQMR raw sequences	iQMRp sequences	Conventional raw sequences	Conventionalp sequences	*χ*^2^-valiue	*P*-value
Tissue edge clarity/sharpness	3.0 (3.0,3.0)	5.0 (5.0,5.0)	4.0 (4.0,4.0)	5.0 (5.0,5.0)	126.201	<0.001
Signal uniformity	3.0 (3.0,3.0)	5.0 (5.0,5.0)	4.0 (4.0,4.0)	5.0 (5.0,5.0)	122.574	<0.001
Fat suppression uniformity	4.0 (3.0,4.0)	4.0 (4.0,4.0)	4.0 (3.0,4.0)	4.0 (4.0,5.0)	51.801	<0.001
Vascular pulsation artifacts	3.0 (3.0,4.0)	3.0 (3.0,4.0)	4.0 (4.0,4.0)	4.0 (4.0,4.0)	63.070	<0.001
Overall image quality	3.0 (3.0,3.0)	5.0 (4.0,5.0)	4.0 (4.0,4.0)	5.0 (5.0,5.0)	112.515	<0.001

iQMR, intelligent quick magnetic resonance; iQMR_p_, iQMR-processed images; Conventional_p_, Conventional-processed images.

**Table 4:** Pairwise comparison results of qualitative assessment.

Evaluation indicators/series	Tissue edge clarity/sharpness	Signal uniformity	Fat suppression uniformity	Vascular pulsation artifacts	Overall image quality
iQMR raw-iQMRp sequences	*P* < 0.05	*P* < 0.05	*P* = 0.945	*P* = 1.000	*P* < 0.05
iQMR raw-Conventional raw sequences	*P* < 0.05	*P* < 0.05	*P* = 1.000	*P* < 0.05	*P* < 0.05
iQMR raw-Conventionalp sequences	*P* < 0.05	*P* < 0.05	*P* < 0.05	*P* < 0.05	*P* < 0.05
iQMRp-Conventional raw sequences	*P* < 0.05	*P* < 0.05	*P* = 1.000	*P* < 0.05	*P* < 0.05
iQMRp-Conventionalp sequences	*P* = 1.000	*P* = 1.000	*P* < 0.05	*P* < 0.05	*P* = 1.000
Conventional raw-Conventionalp sequences	*P* < 0.05	*P* < 0.05	*P* < 0.05	*P* = 1.000	*P* < 0.05

iQMR, intelligent quick magnetic resonance; iQMR_p_, iQMR-processed images; Conventional_p_, Conventional-processed images.

### 3.4 Diagnostic performance

#### 3.4.1 Diagnostic grading results

**Figure 6:**
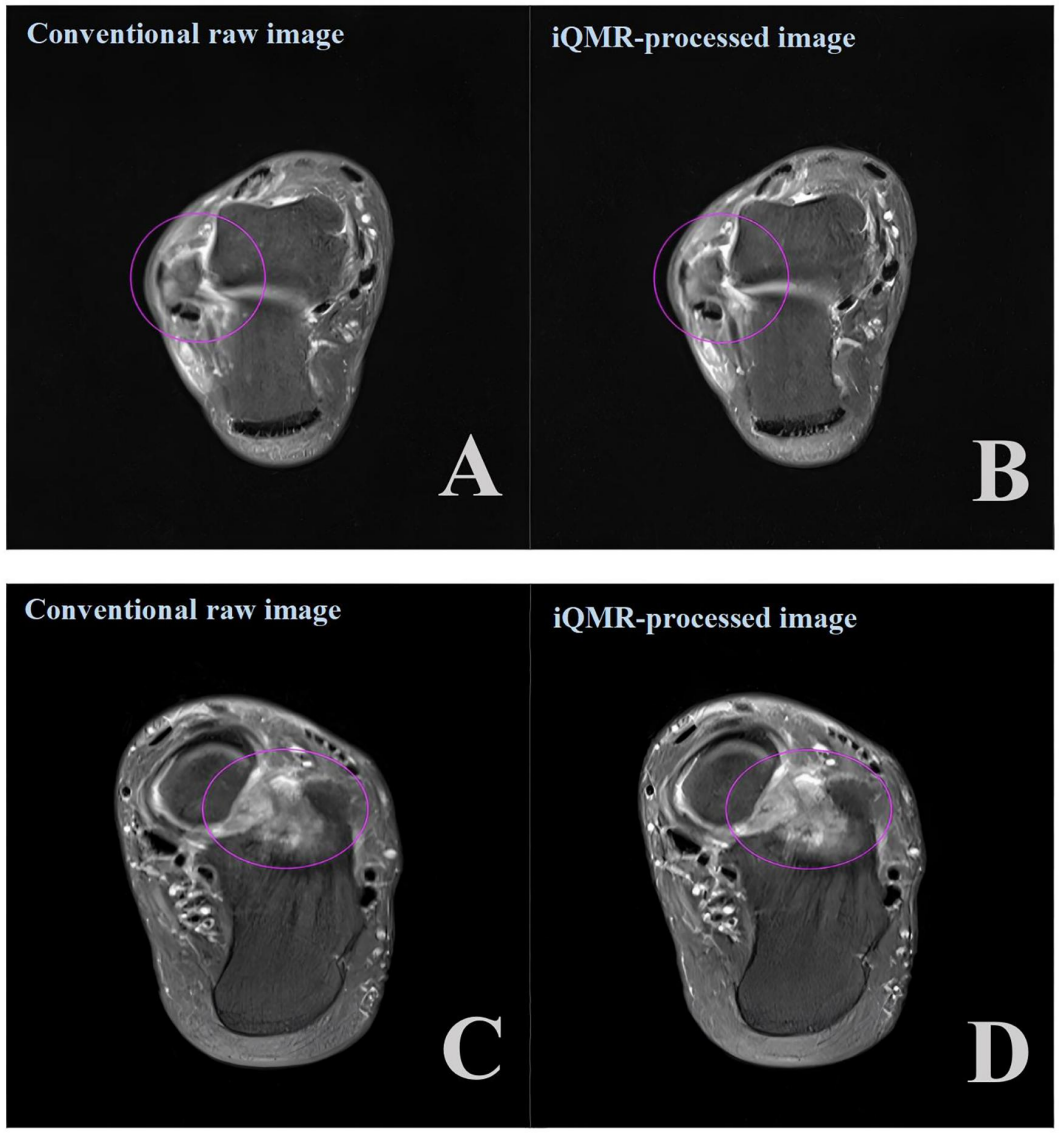
Axial proton density-weighted imaging with fat suppression (PDWI-FS) demonstrating: **(A,B)** ligament injuries (purple circles) using conventional and iQMR protocol; **(C,D)** bone marrow edema (purple circles) using conventional and iQMR protocol. iQMR, intelligent quick magnetic resonance.

The diagnostic accuracy of the four groups of PDWI-FS images for structural injuries of tendons and ligaments was not significantly different (*P* > 0.05, Figures 6A–D). The Kappa of 0.919 (CI: 0.971–0.866)showed a high degree of inter-observer agreement in grading between the two musculoskeletal radiologists.

#### 3.4.2 Diagnostic confidence results

The two radiologists demonstrated excellent agreement in their diagnostic confidence ratings (weighted *κ* = 0.884, CI: 0.932–0.821). Although the median diagnostic confidence for iQMR-processed images was higher than that for conventional raw images across most key structures, the Friedman test revealed no statistically significant differences among the four image sets (*P* > 0.05). Detailed rating results are provided in Supplementary Table S1.

## 4 Discussion

In this study, patients with ankle injuries underwent MRI scans using both iQMR and Conventional sequences. After post-processing at the iQMR workstation, four distinct image sets were generated: (1) iQMR raw images, (2) iQMR-processed images, (3) conventional raw images, and (4) conventional-processed images. These four groups were systematically evaluated for scanning efficiency, image quality, and diagnostic efficacy.

The iQMR-processed sequences reduced acquisition time by 58% compared to Conventional raw sequences. Primarily, shorter scan durations improve patient tolerability, reduce motion artifacts caused by prolonged positioning, and enhance compliance among children, elderly individuals, and patients with claustrophobia, thereby decreasing reliance on sedation and streamlining the examination process (25). Additionally, iQMR technology increases MRI throughput by alleviating appointment backlogs, prioritizing urgent diagnostic cases, and optimizing daily equipment utilization without requiring additional hardware investments (26). Finally, accelerated imaging expands MRI's clinical potential in emergency medicine (e.g., trauma, acute ischemic stroke) and facilitates routine preventive screening protocols.

The iQMR-processed images showed significantly higher SNR and CNR than Conventional raw images (*P* < 0.05), consistent with prior studies. For instance, Liu et al. (17) reported that AI-assisted iterative algorithms improved image quality and scanning efficiency for T1-Weighted Imaging (T1WI), T2-Weighted Imaging (T2WI), and FS-PDWI sequences without compromising diagnostic information. Similarly, Yao et al. (27) validated iQMR's utility in 3D cervical spine MRI, achieving reduced noise and superior SNR and CNR in 2-min scans. However, no significant differences in Achilles tendon SNR were observed between iQMR raw and Conventional raw sequences (*P* > 0.05), likely due to the tendon's dense collagen structure and low water content, which inherently limit signal intensity changes (28–30). This study further validated the agreement and reliability of iQMR post-processing technology for quantitative image quality assessment using Bland-Altman analysis. The analysis demonstrated that despite the significant increase in SNR and CNR values of iQMR-processed images, the differences compared to conventional raw images exhibited no systematic bias, with all data points lying within the limits of agreement. These results substantiate that iQMR technology enhances image quality while maintaining the reliability and reproducibility of measurements, supporting its potential application in clinical quantitative analysis.

The iQMR-processed images scored significantly higher in image quality (*P* < 0.05). However, no significant differences were observed in tissue edge clarity/sharpness, signal uniformity, or overall image quality compared to Conventional-processed images (*P* > 0.05). This discrepancy may attributed to Conventional-processed images already reached diagnostic adequacy thresholds, or the strict double-blind design increased inter-observer variability. Additionally, fat suppression uniformity and vascular pulsation artifacts showed no significant differences across sequences (*P* > 0.05). Despite these findings, the combined benefits of reduced scan time and enhanced objective metrics underscore the clinical value of iQMR-processed images in ankle MRI.

Although no statistical differences emerged in ligaments/tendons injury grading (Schweitzer classification), radiologists reported improved lesion boundary delineation in iQMR-processed images. Enhanced SNR and CNR likely increased radiologists' diagnostic confidence in assessing injury severity, highlighting iQMR technology's potential to optimize clinical decision-making even without altering grading outcomes. Furthermore, this study introduces the novel application of diagnostic confidence ratings in ankle MRI to evaluate the impact of iQMR technology on radiologists' subjective diagnostic confidence. The results demonstrated that, although iQMR-processed images did not yield a statistically significant increase in confidence for most structures, the median ratings were consistently higher than those for conventional raw images. This suggests that by improving the SNR and CNR, iQMR technology may provide ancillary support in delineating complex anatomical boundaries, thereby contributing to a positive trend in diagnostic assessment. This finding indicates that beyond optimizing image quality, iQMR technology possesses potential clinical applicability and may play a supportive role in diagnostic tasks requiring high anatomical detail. Future studies incorporating larger sample sizes and a greater diversity of pathological conditions are warranted to further validate its value in enhancing diagnostic confidence. Moreover, although the two radiologists demonstrated excellent agreement in ligament and tendon injury grading (Schweitzer classification; *κ* = 0.919), we performed an in-depth analysis of cases with discrepant ratings. The results revealed that disagreements were not randomly distributed but were highly concentrated at specific diagnostic thresholds—particularly in differentiating between Schweitzer grade 1 (e.g., thickening or degenerative changes) and grade 2 (partial or complete tear). Imaging findings in such borderline cases often exhibit ambiguity, and diagnostic interpretation depends substantially on SNR, CNR, and the clarity and sharpness of tissue boundaries. The present study found that the iQMR post-processing technology, by improving overall image quality, provides radiologists with richer and clearer diagnostic information. As a result, borderline cases that appear ambiguous and are difficult to interpret on conventional images demonstrate more definitive characteristics on enhanced images, thereby reducing diagnostic uncertainty and minimizing inter-observer variability. This finding further underscores the potential value of iQMR technology in improving diagnostic reliability from the perspective of clinical decision consistency.

This study has several limitations. First, the relatively small sample size (*n* = 46) and the single-center design necessitate future validation through larger-scale, multi-center studies to further confirm the generalizability and robustness of the iQMR technology. The results of this study indicate that iQMR technology offers significant advantages in improving image quality. It is noteworthy that annotated data are often limited in the field of musculoskeletal (MSK) MRI. Recent research has demonstrated that even with scarce annotated data, deep learning methods based on transfer learning can achieve automated detection of joint effusion in knee MRI while maintaining good performance (31). This provides strong support for the application of AI-assisted technologies like iQMR in MSK MRI scenarios involving small datasets, such as ankle MRI. Furthermore, it suggests that future research should place greater emphasis on the robustness of models to domain shifts caused by different scanning protocols. Second, this study evaluated only the PDWI-FS sequence; the performance of iQMR on other crucial sequences, such as T1WI, T2WI, and contrast-enhanced protocols, remains to be systematically investigated. Third, the study was conducted at a 3.0T field strength; the performance and applicability of this technology across different magnetic field strengths (e.g., 1.5T scanners) are currently unknown.

Furthermore, this study primarily focused on the immediate assessment of technical feasibility. All diagnoses were based on imaging evaluation, and the lack of a reference standard, such as surgical pathology or long-term clinical follow-up, may impact the comprehensive validation of injury grading accuracy. Although this study demonstrated the advantage of iQMR in reducing acquisition time, it did not systematically quantify the post-processing time or its overall impact on the end-to-end workflow efficiency from scan initiation to final diagnosis. The improvement in image sharpness and edge clarity achieved by iQMR must ultimately contribute to more accurate feature identification and diagnosis. In challenging tasks such as sperm morphology classification, deep learning has not only achieved high classification accuracy but, more importantly, has utilized interpretability techniques (e.g., Grad-CAM) to visualize the decision-making process, thereby establishing an intuitive link between morphological features and classification outcomes (32). Inspired by this approach, our future work will incorporate similar explainable analyses (e.g., generating attention maps) to visually demonstrate how iQMR reconstruction enhances the visualization of anatomically critical features—such as ligamentous fibers, subtle tendon tears, and cartilage surfaces—that are essential for diagnosis. This will more directly link the image quality metrics to the underlying mechanisms that boost diagnostic confidence. Additionally, the study did not explore the technology's influence on ultimate clinical endpoints, such as long-term patient outcomes, clinical decision-making, or the reduction of repeat scan rates. Finally, the current validation dataset lacks sufficient diversity in pathology types and injury severity distributions, preventing a systematic evaluation of the algorithm's consistency in visualizing lesions from different tissue origins or varying degrees of severity.

Future research will aim to build richer, multi-parametric, multi-field-strength datasets through multi-center collaboration and to systematically collect surgical pathology and follow-up data. This will enable a comprehensive assessment of the clinical reliability, long-term benefits, and value of iQMR technology in optimizing the overall diagnostic workflow.

In summary, the iQMR technology can significantly shorten ankle MRI scan time, reduce motion artifacts, and improve diagnostic accuracy without sacrificing image quality, suggesting its potential for clinical utility.

## Data Availability

The original contributions presented in the study are included in the article/Supplementary Material, further inquiries can be directed to the corresponding authors.

## References

[1] Foreman SC Neumann J Han J Harrasser N Weiss K Peeters JM Deep learning-based acceleration of compressed sense MR imaging of the ankle. Eur Radiol. (2022) 32(12):8376–85. 10.1007/s00330-022-08919-9 35751695 PMC9705492

[2] Jiang XT Cheng TX Li FF Yuan Y Jiang L Wei J Application of artificial intelligence compressive sensing technology in MRI of the ankle joint. Chin J Med Imaging. (2024) 32(11):1164–9. 10.3969/j.issn.1005-5185.2024.11.013

[3] Zhang XY Ma PQ Yuan YS Wang ZQ Peng B Zhang ZX. The value of compressed sensing 3D MRI sagittal T2 weighted imaging-spectral attenuatedin-version recovery to display the anterior talofibular ligament. Chin J Magn Reson Imaging. (2023) 14(06):71–4; +81. 10.12015/issn.1674-8034.2023.06.011

[4] Thomas A Fredette R Han G Curtin P Swart E. Can lateral x-rays reliably determine which posterior malleolus ankle fractures need a CT? Foot Ankle Spec. (2024) 17(6):585–91. 10.1177/19386400221128159 36217982

[5] Tuncer K Topal M Tekin E Sade R Pirimoglu RB Polat G. The new ultralow dose CT protocol for the diagnosis of fractures of the ankle: a prospective comparative study with conventional CT. J Orthop Surg (Hong Kong). (2020) 28(3):2309499020960238. 10.1177/2309499020960238 32985384

[6] Feng L Benkert T Block KT Sodickson DK Otazo R Chandarana H. Compressed sensing for body MRI. J Magn Reson Imaging. (2017) 45(4):966–87. 10.1002/jmri.25547 27981664 PMC5352490

[7] Wang J Zhou Y. Advances in imaging studies of chronic ankle instability. Chin J Sports Med. (2023) 42(05):395–400. 10.3969/j.issn.1000-6710.2023.05.010

[8] Garwood ER Recht MP White LM. Advanced imaging techniques in the knee: benefits and limitations of new rapid acquisition strategies for routine knee MRI. AJR Am J Roentgenol. (2017) 209(3):552–60. 10.2214/AJR.17.18228 28639870

[9] Wang Q Wu YB Pan SN Zhang GX. Research progresses in 3D-MRI on ankle cartilage injuries. Chin J Med Imaging Technol. (2024) 40(05):791–4. 10.13929/j.issn.1003-3289.2024.05.033

[10] Fritz J Guggenberger R Del Grande F. Rapid musculoskeletal MRI in 2021: clinical application of advanced accelerated techniques. AJR Am J Roentgenol. (2021) 216(3):718–33. 10.2214/AJR.20.22902 33534618

[11] Roh S Park JI Kim GY Yoo HJ Nickel D Koerzdoerfer G Feasibility and clinical usefulness of deep learning-accelerated MRI for acute painful fracture patients wearing a splint: a prospective comparative study. PLoS One. (2023) 18(6):e0287903. 10.1371/journal.pone.0287903 37379272 PMC10306199

[12] Wang Q Xing X Zhang Z Ji X He S Yang Y Added value of 3D fast-field-echo (FRACTURE) sequences for cervical spondylosis diagnosis: a prospective multi-reader non-inferiority study. Insights Imaging. (2025) 16(1):114. 10.1186/s13244-025-01997-5 40459683 PMC12133650

[13] Zheng G Fu J Wang Z Li W Li A Yu D. AI-assisted compressed sensing MRI improves imaging quality in rectal cancer: a comparative study with conventional acceleration techniques. Quant Imaging Med Surg. (2025) 15(3):2547–60. 10.21037/qims-24-1317 40160648 PMC11948424

[14] Ikeda H Ohno Y Murayama K Yamamoto K Iwase A Fukuba T Compressed sensing and parallel imaging accelerated T2 FSE sequence for head and neck MR imaging: comparison of its utility in routine clinical practice. Eur J Radiol. (2021) 135:109501. 10.1016/j.ejrad.2020.109501 33395594

[15] Won-Joon D Sunghun S Yoseob H Chul YJ Hong CS Sung-Hong P. Reconstruction of multicontrast MR images through deep learning. Med Phys. (2020) 47(3):983–97. 10.1002/mp.14006 31889314

[16] Hu JY Yang XX. The application value of iterative image reconstruction algorithm based on machine learning in MRI scanning in skeletal system. J Clin Res. (2023) 40(12):1846–9. 10.3969/j.issn.1671-7171.2023.12.007

[17] Liu H Chen Y Zhang M Bu H Lin F Chen J Feasibility of knee magnetic resonance imaging protocol using artificial intelligence-assisted iterative algorithm protocols: comparison with standard MRI protocols. Front Med (Lausanne). (2024) 11:1480196. 10.3389/fmed.2024.1480196 39507702 PMC11537882

[18] Johnson PM Recht MP Knoll F. Improving the speed of MRI with artificial intelligence. Semin Musculoskelet Radiol. (2020) 24(1):12–20. 10.1055/s-0039-3400265 31991448 PMC7416509

[19] Kanemaru N Takao H Amemiya S Abe O. The effect of a post-scan processing denoising system on image quality and morphometric analysis. J Neuroradiol. (2022) 49(2):205–12. 10.1016/j.neurad.2021.11.007 34863809

[20] Wen SL Zhou CY Zeng ZS. Application of deep learning reconstruction techniques for accelerated knee MRI. J Imaging Res Med Appl. (2024) 8(17):78–80. 10.3969/j.issn.2096-3807.2024.17.024

[21] Wu Y Xu M Liu J Wang RP Zeng XC. The application value of intelligent quick magnetic resonance technology in head MRI scan. J Clin Radiol. (2024) 43(03):337–40.

[22] Xie XL Wang JW Yan XH He L Zhou QL Chen C Application value of intelligent quick magnetic resonance technology in supraspinatus tendon injuries. Chin J Magn Reson Imaging. (2024) 15(10):148–52; +64. 10.12015/issn.1674-8034.2024.10.025

[23] Xu M Wu Y Liu J Wang RP Xu R Zeng XC. Application value of intelligent quick magnetic resonance technique in magnetic resonance scanning ofcervical vertebra. Chin J Magn Reson Imaging. (2023) 14(10):111–5. 10.12015/issn.1674-8034.2023.10.019

[24] Schweitzer ME Tran D Deely DM Hume EL. Medial collateral ligament injuries: evaluation of multiple signs, prevalence and location of associated bone bruises, and assessment with MR imaging. Radiology. (1995) 194(3):825–9. 10.1148/radiology.194.3.7862987 7862987

[25] Foti G Longo C. Deep learning and AI in reducing magnetic resonance imaging scanning time: advantages and pitfalls in clinical practice. Pol J Radiol. (2024) 89:e443–51. 10.5114/pjr/192822 39444654 PMC11497590

[26] Wang Q Zhao W Xing X Wang Y Xin P Chen Y Feasibility of AI-assisted compressed sensing protocols in knee MR imaging: a prospective multi-reader study. Eur Radiol. (2023) 33(12):8585–96. 10.1007/s00330-023-09823-6 37382615 PMC10667384

[27] Yao H Jia B Pan X Sun J. Validation and feasibility of ultrafast cervical spine MRI using a deep learning-assisted 3D iterative image enhancement system. J Multidiscip Healthc. (2024) 17:2499–509. 10.2147/JMDH.S465002 38799011 PMC11128255

[28] Fullerton GD Rahal A. Collagen structure: the molecular source of the tendon magic angle effect. J Magn Reson Imaging. (2007) 25(2):345–61. 10.1002/jmri.20808 17260393

[29] Pierre-Jerome C Moncayo V Terk MR. MRI Of the achilles tendon: a comprehensive review of the anatomy, biomechanics, and imaging of overuse tendinopathies. Acta Radiol. (2010) 51(4):438–54. 10.3109/02841851003627809 20380605

[30] Szaro P Nilsson-Helander K Carmont M. MRI Of the achilles tendon-A comprehensive pictorial review. Part one. Eur J Radiol Open. (2021) 8:100342. 10.1016/j.ejro.2021.100342 33850971 PMC8039565

[31] Iqbal I Shahzad G Rafiq N Mustafa G Ma J. Deep learning-based automated detection of human knee joint’s synovial fluid from magnetic resonance images with transfer learning. IET Image Process. (2020) 14(10):1990–8. 10.1049/iet-ipr.2019.1646

[32] Iqbal I Mustafa G Ma J. Deep learning-based morphological classification of human sperm heads. Diagnostics (Basel). (2020) 10(5):325. 10.3390/diagnostics10050325 32443809 PMC7277990

