# Supplementary material for: Feasibility of artificial intelligence-assisted fast magnetic resonance imaging technology in the ankle joint injury: a comparison of the proton density-weighted image
Source: Front Radiol. 2025 Oct 24;5:1673619. doi: 10.3389/fradi.2025.1673619 (PMC12592152; doi:10.3389/fradi.2025.1673619)
Supplement: Supplementary file 1 [file Table1.docx]

**Comparison of Diagnostic Confidence Scores for Key Structures Under Two Different Sequences (n=46)**

| Anatomical Structure | Conventional raw images  (radiologist-A) | Conventional raw images  (radiologist-B) | iQMR-processed images  (radiologist-A) | iQMR-processed images  (radiologist-B) | P-value |
| --- | --- | --- | --- | --- | --- |
| Anterior Talofibular Ligament | 4 (3 , 4) | 4 (3 , 4) | 5 (4 , 5) | 5 (4 , 5) | P>0.05 |
| Posterior Talofibular Ligament | 4 (3 , 4) | 4 (3 , 5) | 5 (4 , 5) | 5 (4 , 5) | P>0.05 |
| Calcaneofibular Ligament | 3 (2 , 4) | 4 (3 , 5) | 4 (3 , 5) | 4 (3 , 5) | P>0.05 |
| Achilles Tendon | 4 (3 , 5) | 4 (3 , 5) | 4 (3 , 5) | 5 (4 , 5) | P>0.05 |
| Posterior Tibial Tendon | 3 (2 , 4) | 3 (2 , 4) | 4 (3 , 5) | 4 (3 , 5) | P>0.05 |
| Flexor Digitorum Longus Tendon | 3 (2 , 4) | 4 (3 , 5) | 4 (3 , 5) | 4 (3 , 5) | P>0.05 |
| Flexor Hallucis Longus Tendon | 3 (2 , 4) | 3 (2 , 4) | 4 (3 , 5) | 4 (3 , 5) | P>0.05 |
| Peroneus Brevis Tendon | 3 (2 , 4) | 3 (2 , 4) | 4 (3 , 5) | 4 (3 , 5) | P>0.05 |
| Peroneus Longus Tendon | 3 (2 , 4) | 3 (2 , 4) | 4 (3 , 5) | 4 (3 , 5) | P>0.05 |
